# Diagnostic accuracy of D-dimer in periprosthetic joint infection: a diagnostic meta-analysis

**DOI:** 10.1186/s13018-020-01853-w

**Published:** 2020-08-17

**Authors:** Haitao Zhang, Xiaobo Sun, Pengfei Xin, Xingyang Zhu, Ke Jie, Houran Cao, Wenjun Feng, Yuqing Zeng, Yan Lv, Jinlun Chen, Jie Li, Jianchun Zeng, Yirong Zeng

**Affiliations:** 1 The First Clinical Medical School, Guangzhou University of Chinese Medicine, Jichang Road 12#, District Baiyun, Guangzhou, Guangdong China; 2 Department of Orthopaedics, The First Affiliated Hospital of Guangzhou University of Chinese Medicine, Jichang Road 16#, District Baiyun, Guangzhou, 510405 Guangdong China; 3 The First Affiliated Hospital of Nanchang University, 17 Yongwai Street, Nanchang, 330006 China; 4 Ganzhou Hospital of Traditional Chinese Medicine, Xijin Road 16#, District Zhanggong, Ganzhou, Jiangxi China

**Keywords:** Periprosthetic joint infection, D-dimer, Diagnosis, Meta-analysis

## Abstract

**Background:**

Periprosthetic joint infection (PJI) is one of the most devastating complications after total joint replacement (TJA). Up to now, the diagnosis of PJI is still in a dilemma. As a novel biomarker, whether D-dimer is valuable in the diagnosis of PJI remains controversial. This meta-analysis attempts to determine the diagnostic accuracy of D-dimer in PJI.

**Methods:**

Relevant literature was retrieved from PubMed, Embase, Web of Science, and Cochrane Library (from database establishment to April 2020). Literature quality was evaluated using Revman (version 5.3). The random effect model was used in the Stata version 14.0 software to combine sensitivity, specificity, likelihood ratio (LR), diagnostic odds ratio (DOR), summary receiver operating characteristic (SROC) curve, and area under SROC (AUC) to evaluate the diagnostic value of overall D-dimer for PJI. Meta regression and subgroup analysis were performed according to the threshold, the study design, the sample size, the diagnostic gold standard, the country of study, and the type of sample.

**Results:**

A total of 9 studies were included in this study, including 1592 patients. The pooled sensitivity and specificity of D-dimer for PJI diagnosis are 0.82 (95% CI, 0.72~0.89) and 0.73 (95% CI, 0.58~0.83), respectively. The pooled positive likelihood ratio (PLR) and negative likelihood ratio (NLR) were 2.99 (95% CI, 1.84~4.88) and 0.25 (95% CI, 0.15~0.41), respectively. The pooled AUC and diagnostic odds ratios were 0.85 (95% CI, 0.82~0.88) and 12.20 (95% CI, 4.98~29.86), respectively.

**Conclusion:**

D-dimer is a promising biomarker for the diagnosis of PJI, which should be used in conjunction with other biomarkers or as an adjunct to other diagnostic methods to enhance diagnostic performance.

## Introduction

Periprosthetic joint infection (PJI) is regarded as a destructive complication after total joint replacement (TJA) [1], accounting for approximately 15% and 25% of the failure factors of total hip and knee arthroplasty, respectively [2, 3]. With the extension of the average life span of human beings, there are an increasing number of TJA operations, and the resulting number of PJI patients has also been increasing year by year [4]. The existence of PJI seriously reduces the quality of life of patients, exacerbates the difficulty of treatment for orthopedic surgeons, and aggravates the financial burden of the national health system [5–7]. Moreover, it is extremely difficult to accurately diagnose PJI, due to the atypical symptoms of many PJI patients [8]. Many scholars jointly established the American Academy of Orthopedic Surgeon (AAOS)’s guidelines, Musculoskeletal Infection Society (MSIS), and International Consensus Meeting (ICM) diagnostic criteria in 2010, 2011, and 2013 respectively, which have been widely used [9–11]. However, currently, there is still no diagnostic standard or indicators that can achieve 100% diagnostic accuracy, and some standards including synovial fluid detection are invasive, expensive, and inconvenient to obtain [12]. Given this situation, there is an urgent demand for joint surgeons to look for diagnostic markers with efficiency, cheapness, and convenience [13]. D-dimer is a fibrin degradation product formed by fibrinolysis of fibrin clots, which reflects the state of blood coagulation and increases in systemic or local infections, thrombosis, and neoplastic diseases [14–16]. Based on this principle, Shahi et al. and Li et al. speculated that D-dimer in PJI patients might serve as a neoteric diagnostic biomarker [17, 18]. Nevertheless, their findings are contradictory. There is also no agreement on the conclusions of the other similar studies. Therefore, we conducted a systematic review and meta-analysis of these literatures to study the diagnostic value of D-dimer in PJI.

## Methods

Our study is carried out strictly in accordance with the criterion of the preferred report items of systematic review and meta-analysis report. The research scheme is determined by all the authors, and the steps of literature retrieval, literature quality evaluation, data statistics, result merging, and report writing are completed in turn.

### Retrieval strategy

Under the guidance of the Cochrane Review method, two authors (Haitao Zhang and Xingyang Zhu) search online databases such as PubMed, Embase, Web of Science, and Cochrane Library. The search subject words and Mesh words are as follows: “periprosthetic joint infection” or “prosthesis-related infections” stands for disease, “D-dimer” or “D-dimer fibrin” or “D-dimer fragments” or “fibrin fragment D1 dimer” or “fibrin fragment DD” or “fibrin fragment D-dimer” represents target index. The range of retrieval dates is from the establishment of the database to April 2020. When searching, we only include English literature. After database screening, we manually searched some of the references included in the literature to obtain the valuable literature for this study.

### Study selection

The title, abstract, or full text of all search results are reviewed by two censors (Haitao Zhang and Xiaobo Sun) in detail. When there is still a disagreement between the two examiners after reading the full text, it will be left to Professor Yirong Zeng to make the final decision. The inclusion criteria of the literature are as follows: (1) using D-dimer as an index for the diagnosis of PJI, (2) have integrated data (including true positive, false negative, false positive, and true negative) to construct a 2 × 2 table, and (3) there is a definite gold standard such as Musculoskeletal Infection Society (MSIS) or International consensus on infection (ICM) to compare the diagnostic accuracy with D-dimer.

### Data extraction and quality assessment

The following data extraction and literature quality evaluation are completed by Pengfei Xin and Ke Jie, two researchers who are familiar with the knowledge of statistics, back to back, and the extracted data are input into a table in Excel. The data include the following: the author of the study, the year, the country or region in which the article was published, the design type of the study, the number of cases, sex ratio, patient’s age and BIM index, the gold standard used in the study, the detection method, and the cutoff value of D-dimer. In addition, the diagnostic accuracy of D-dimer (AUC) and the true positive, false positive, true negative, and false negative data used to construct 2 × 2 table were also recorded in detail.

The Quality Assessment of Diagnostic Accuracy Studies (QUADAS-2) in the Revman (version5.3) software was used to evaluate the quality of all the literature included in the study. QUADAS-2 is an updated version of the original QUADAS, including four aspects of patient selection, index test, reference standard, and flow and timing, which has a more accurate bias level and applicability to the original research than the original QUADAS.

### Statistical analysis

All data analysis and picture production are carried out by using the commands in the Stata14.0 software. The bivariate random effect model was selected to analyze the tp, fp, fn, and tn values of 2 × 2 table extracted in the study and to test the heterogeneity. The sensitivity, specificity, positive likelihood ratio (PLR), negative likelihood ratio (NLR), diagnostic score, and diagnostic odds ratio (DOR) were obtained after integration. Among them, the higher the value of DOR, the higher the diagnostic value.

Additionally, the summary receiver operating characteristic (SROC) was drawn by the Midas command, and the area under the curve (AUC) was calculated. AUC represents the diagnostic accuracy of D-dimer.

The heterogeneity is expressed as the inconsistency index (*I*^2^) statistic, the smaller the *I*^2^, the smaller the heterogeneity. When *I*^2^ is 75%, 50%, and 25% respectively, it corresponds to large, medium, and small literature heterogeneity, respectively. If the heterogeneity is large, meta-regression and subgroup analysis are performed to identify the source of heterogeneity. We believe that the variables that may affect the heterogeneity are the type of study design, the threshold used in the study, the number of cases, the index of diagnostic gold, the sample type, and the country or region in which the literature is published.

In order to definitely judge the publication bias, the funnel chart (Deeks’ funnel plot) was drawn. Besides, the change of the diagnostic value of D-dimer on the incidence of PJI can be clearly shown by drawing a Fagan plot diagram.

## Results

### Search results and study characteristics

**Fig. 1:**
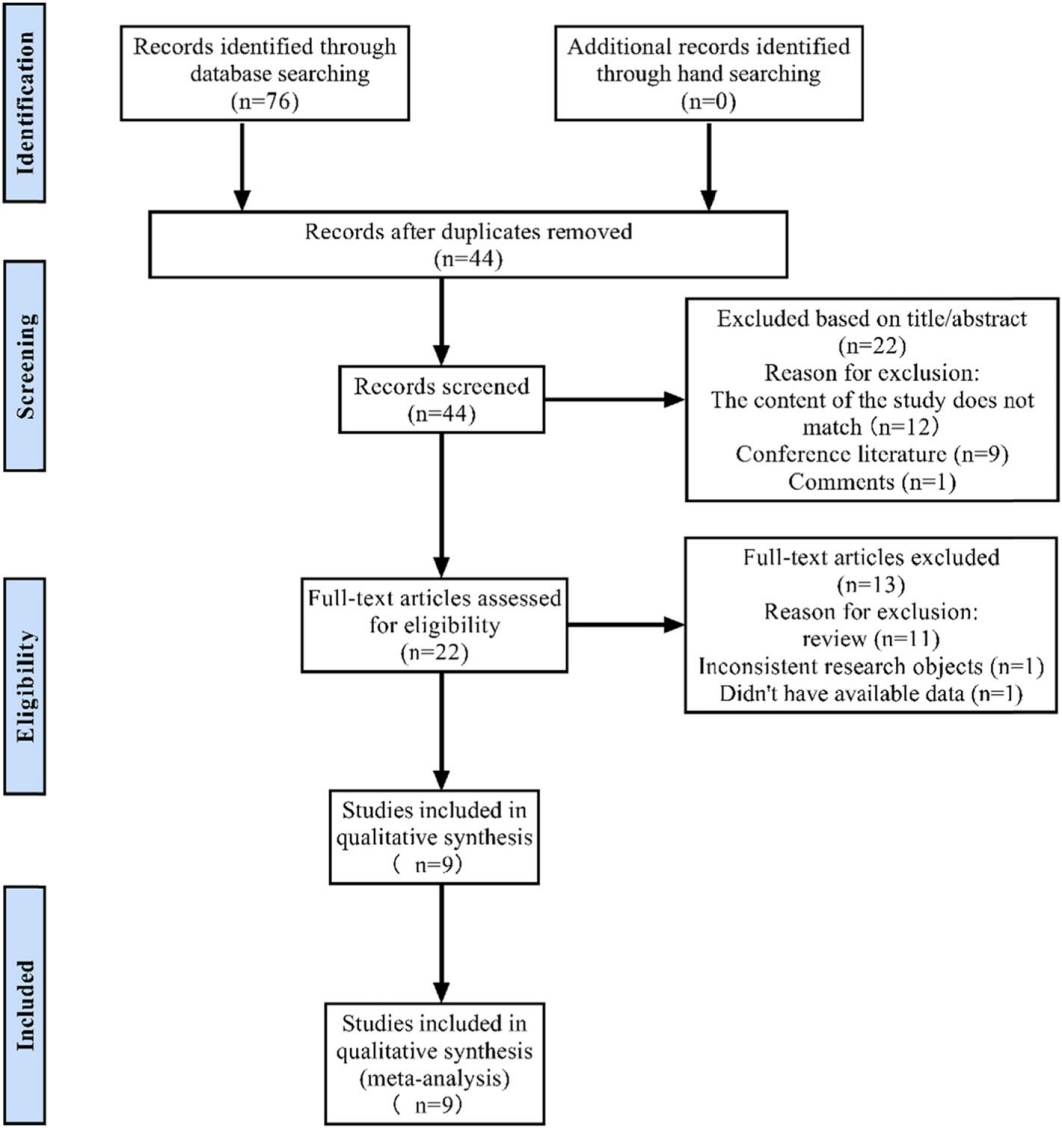
Flow diagram for study selection

The details of the literature screening process are shown in Fig. 1. Through preliminary search, a total of 76 articles were identified in three online databases. Among them, 19 articles were obtained by PubMed, 24 articles were obtained by Web of Science, and 33 articles were obtained by Embase. After 32 repeated literatures are excluded, there are 44 remaining ones. Then 22 articles were excluded by reading titles and abstracts, including 12 inconsistent research contents, 9 reviews, and 1 conference literature. Of the remaining 22 articles, 13 were deleted after reading the full text, including 1 literature with unavailable data, 1 literature with inconsistent research objects, and 11 reviews. Finally, a total of 9 articles were included in this study for meta-analysis.

A total of 1592 patients were enrolled in nine studies [12, 17–24], including 1061 patients with non-PJI and 531 patients with confirmed PJI. All of these patients underwent knee or hip arthroplasty, but no other parts of total joint arthroplasty were performed. Five studies were retrospective and four were prospective. Seven of the studies were from China and two were performed in the USA. Seven studies regarded MSIS as the “gold standard” for the diagnosis of PJI, and only 2 studies adopted ICM as “the gold” standard for diagnosis. Table 1 shows the detailed characteristics of all the studies. Table 2 summarizes the data extraction results of each study (2 × 2 table).

**Table 1:** Characteristics of the studies in meta-analysis for the diagnosis of PJI applying D-dimer

Study	Year	Country	Study design	Gender (M/F)	Median age^*^	BMI^*^	Detection method	Cutoff values	Gold standard
Pannu et al. [21]	2020	USA	R	62/49	68/70	30.5/30.5	NA	850 ng/ml	ICM
Hu et al. [12]	2020	China	R	37/40	65.57/60.78	NA	Immunoturbidimetric assay	0.955 μg/ml	MSIS
Xu et al. [24]	2019	China	R	NA	NA	NA	NA	1.02 mg/L FEU	MSIS
Xiong et al. [23]	2019	China	P	54/26	59.76/65.42	22.87/25.07	Immunoturbidimetric assay	756 ng/ml	MSIS
Qin et al. [22]	2019	China	P	53/69	64.66/65.89	23.84/22.12	NA	1170 ng/ml	MSIS
Li et al. [17]	2019	China	R	470/95	61.3/63.7	25.15/25.01	STA-R Evolution analyzer	1.25 mg/ml	ICM
Shahi et al. [18]	2017	USA	P	101/94	NA	NA	NA	850 ng/ml	MSIS
Fu et al. [19]	2019	China	P	9/21	65.47/65.60	24.67/25.72	NA	850 ng/ml	MSIS
Huang et al. [20]	2019	China	R	NA	69.27/64.94	NA	NA	850 ng/ml	MSIS

^*^The values were given as the number with non-PJI/PJI

*P* prospective study, *R* retrospective study, *NA* not applicable

**Table 2:** Data extracted for the construction of 2 × 2 table

Author	Year	TP	FP	FN	TN	Total
Pannu et al.	2020	47	42	2	20	111
Hu et al.	2020	35	4	5	33	77
Xu et al.	2019	88	93	41	96	318
Xiong et al.	2019	21	11	5	43	80
Qin et al.	2019	51	17	4	50	122
Li et al.	2019	61	165	35	305	566
Shahi et al.	2017	51	10	6	128	195
Fu et al.	2019	10	6	5	9	30
Huang et al.	2019	22	14	9	56	101

*TP* true positive, *FP* false positive, *FN* false negative, *TN* true negative

### Quality assessment and publication biases

**Fig. 2:**
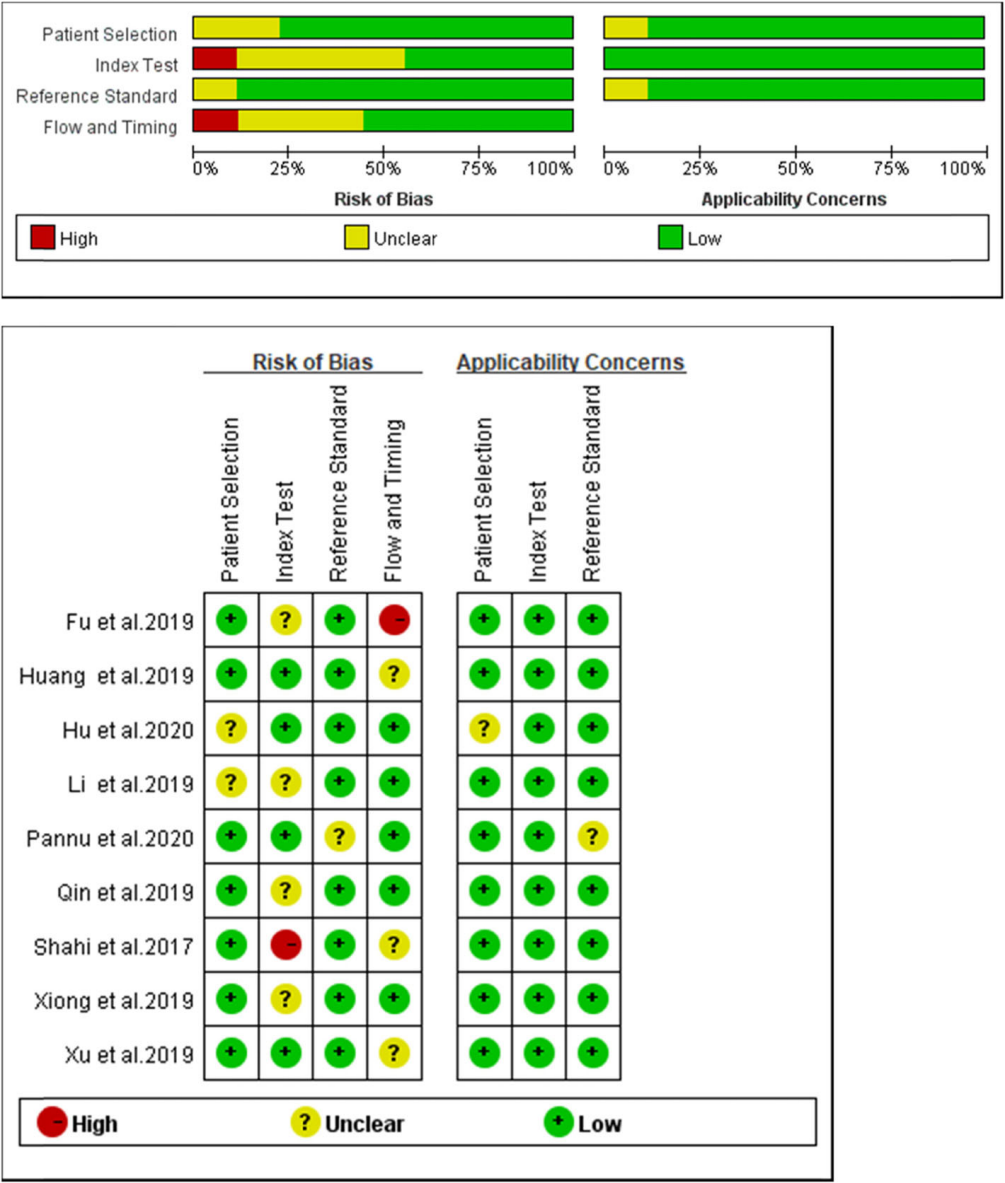
Quality assessment of included studies based on QUADAS-2 tool criteria

**Fig. 3:**
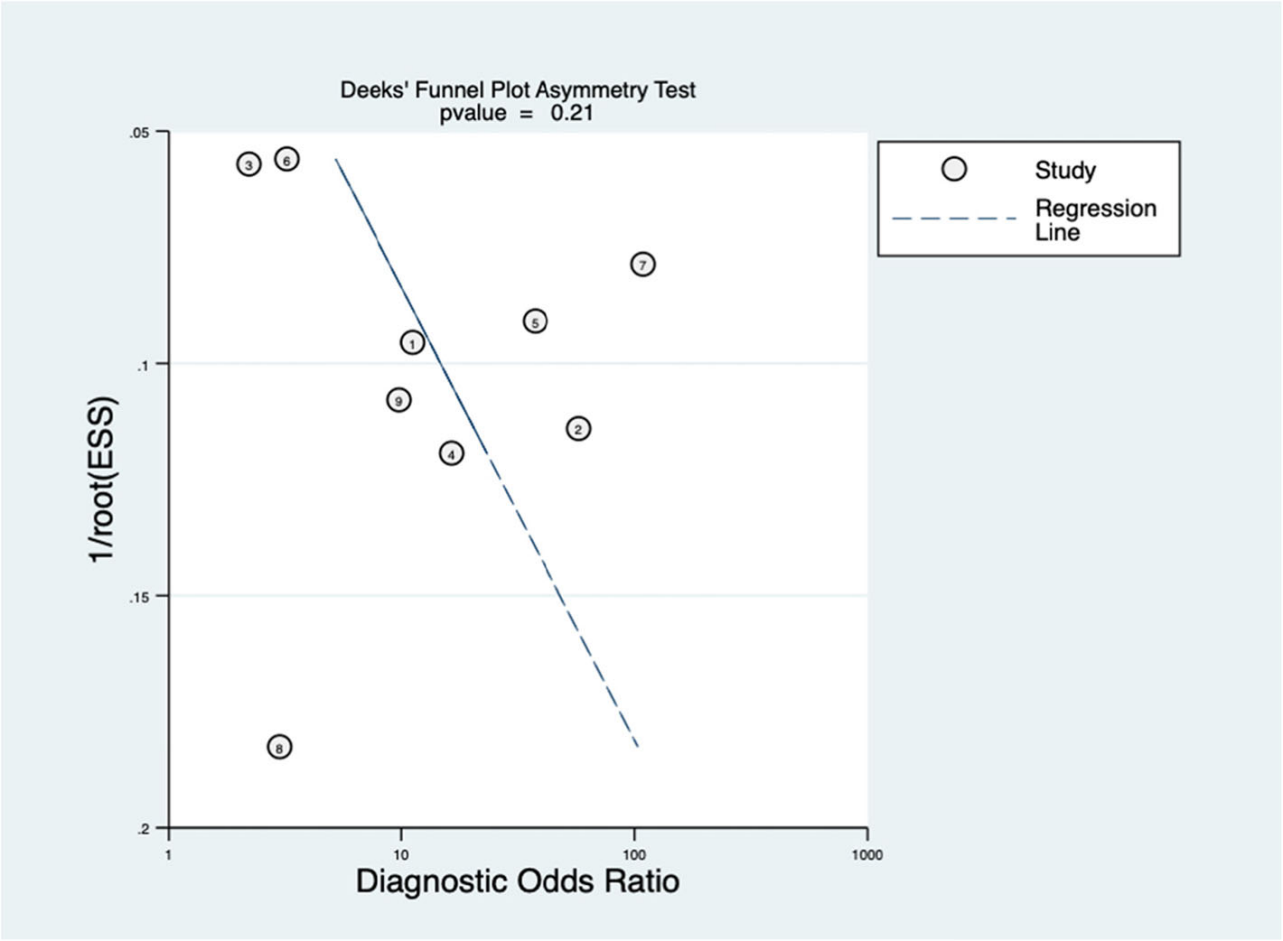
Funnel plot for publication bias assessment of included studies

The quality assessment results of 9 studies using the QUADAS-2 scale are shown in Fig. 2. The figure shows that there are two “high risks,” and the rest are “unclear” or “low risk.” Generally speaking, the quality of literature is in the upper-middle level. By drawing the Deeks’ funnel plot diagram, we can clearly see that there is a slightly symmetrical trend on both sides, and the *p* = 0.21, much greater than 0.05. Therefore, there is no conspicuous publication bias (Fig. 3).

### Threshold and diagnostic accuracy of D-dimer for PJI

**Fig. 4:**
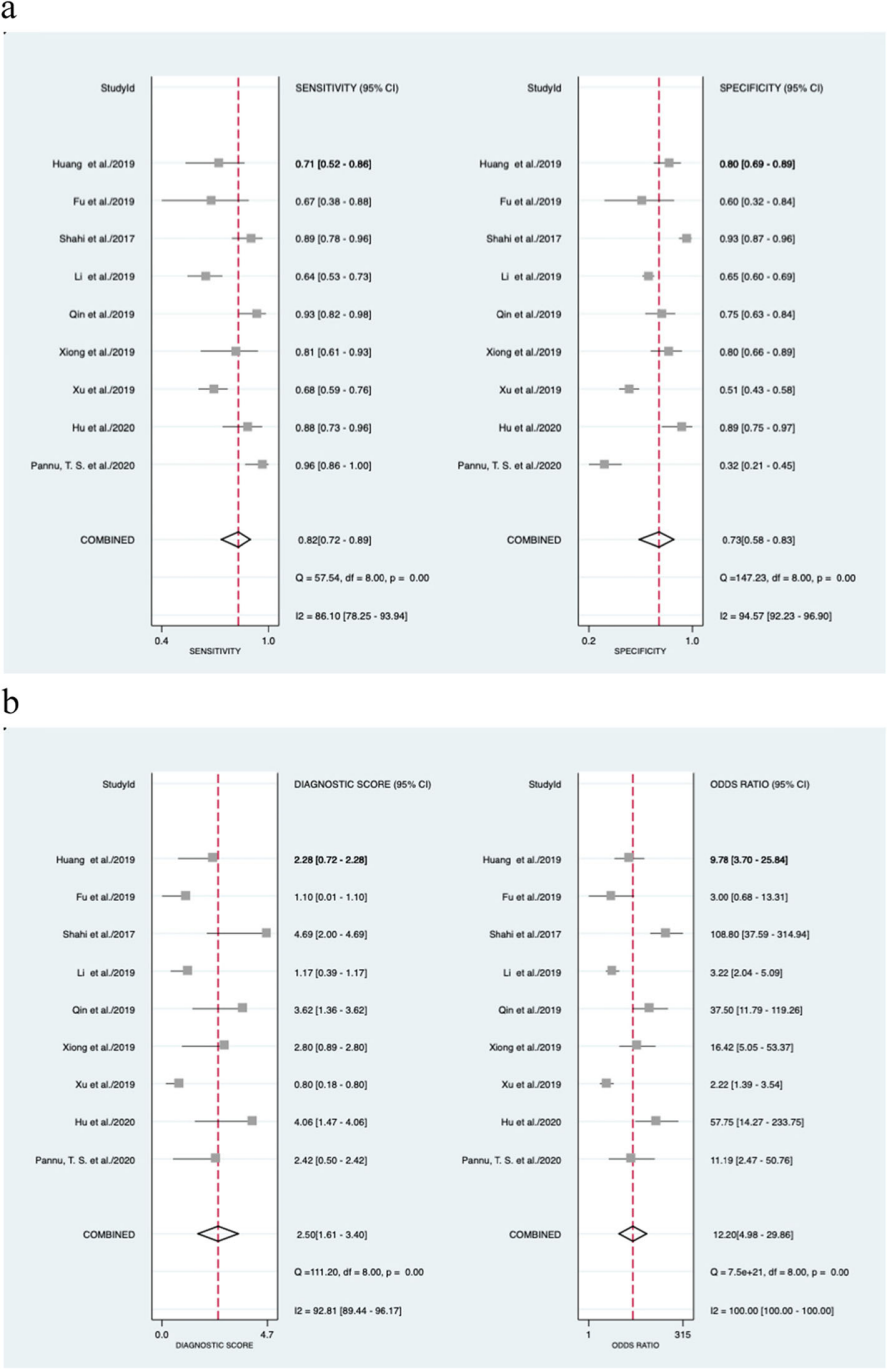
Forest plot of D-dimer for PJI. **a** Pooled sensitivity and specificity. **b** Pooled diagnostic score and diagnostic odds ratio

**Fig. 5:**
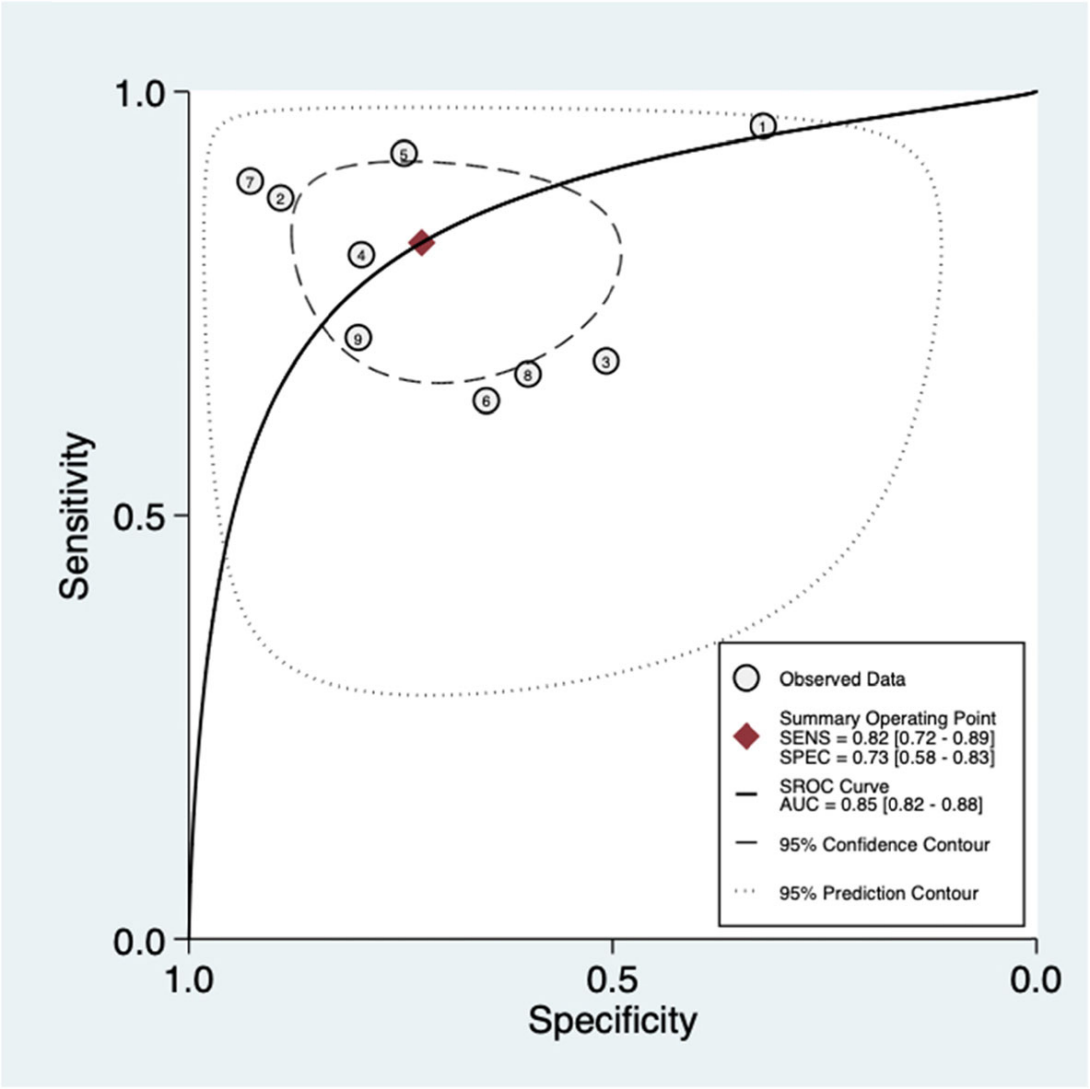
SROC curve of included studies

Regarding the cutoff value, four studies adopted the common threshold (850 ng/ml), while different thresholds were adopted by the remaining 5 studies. The forest diagram shows that the pooled sensitivity and specificity of D-dimer for PJI diagnosis are 0.82 (95% CI, 0.72~0.89) and 0.73 (95% CI, 0.58~0.83), respectively (Fig. 4a). The sensitivity and specificity of the corresponding *I*^2^ statistics were 86.10 (95% CI, 78.25~93.94) and 94.57 (95% CI, 92.23–96.90), respectively. Thus, it can be seen that there is a great heterogeneity among the studies. The pooled diagnostic score and diagnostic odds ratio were 2.50 (95% CI, 1.61~3.40) and 12.20 (95% CI, 4.98~29.86), respectively (Fig. 4b). The area under SROC (AUC) is 0.85 (95% CI, 0.82~0.88) (Fig. 5).

### Evaluation of the clinical utility

**Fig. 6:**
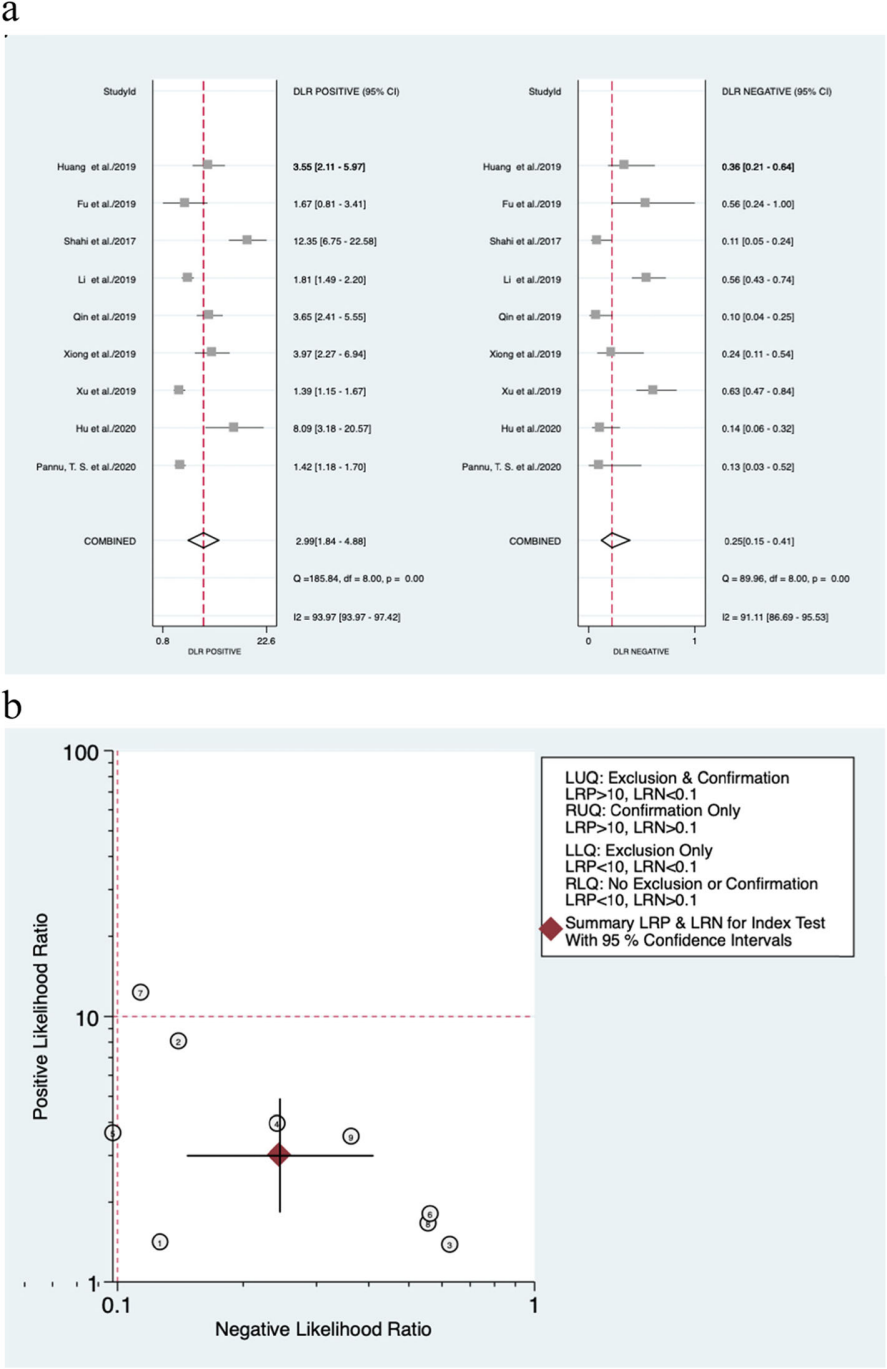
Forest plots of likelihood ratio (**a**) and likelihood ratio scatter diagrams (**b**)

**Fig. 7:**
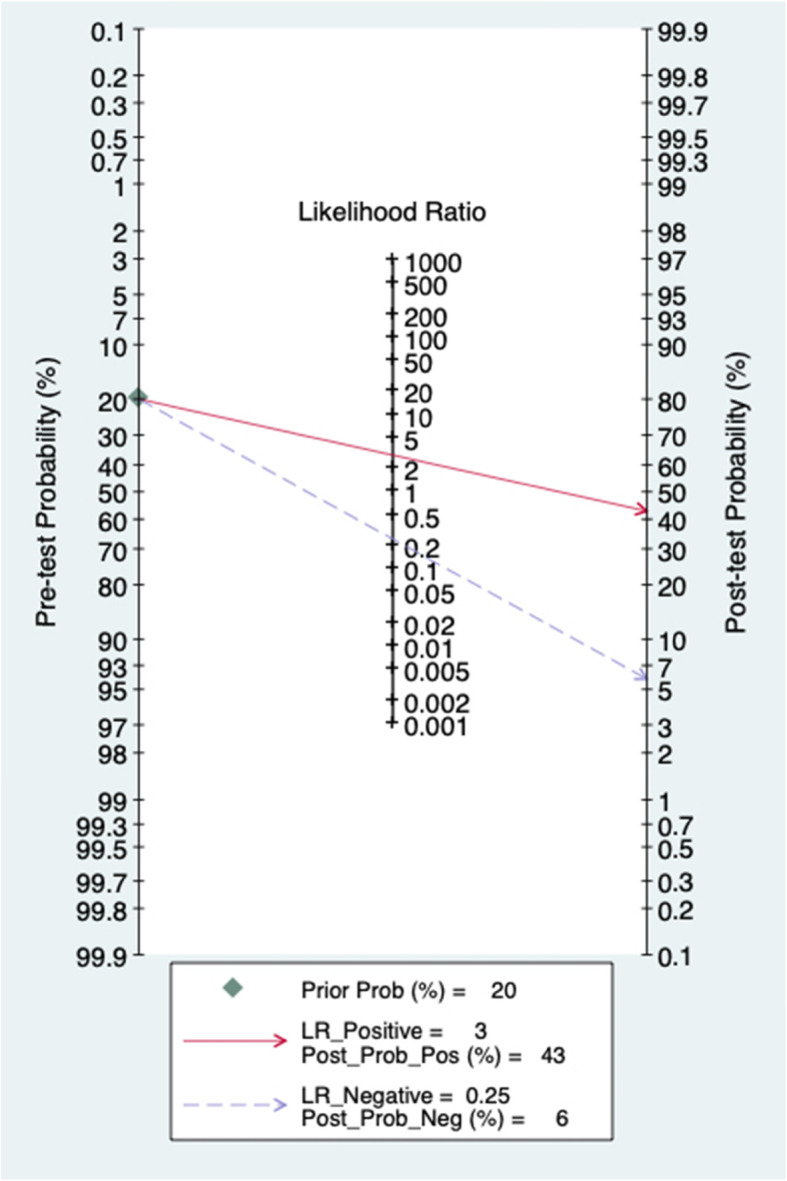
Fagan’s nomogram of the D-dimer for diagnosis of PJI

The pooled PLR and NLR of D-dimer for PJI diagnosis were 2.99 (95% CI, 1.84~4.88) and 0.25 (95% CI, 0.15~0.41), respectively (Fig. 6). According to previous studies, the incidence of PJI accounts for approximately 20% of revision arthroplasty. Hence, 0.2 pretest probabilities was selected to calculate the posttest probability through the likelihood ratio and the pretest probability [25]. The posttest probability of PJI was 6%, indicating negative D-dimer results (Fig. 7).

### Meta-regression and subgroup analysis

**Fig. 8:**
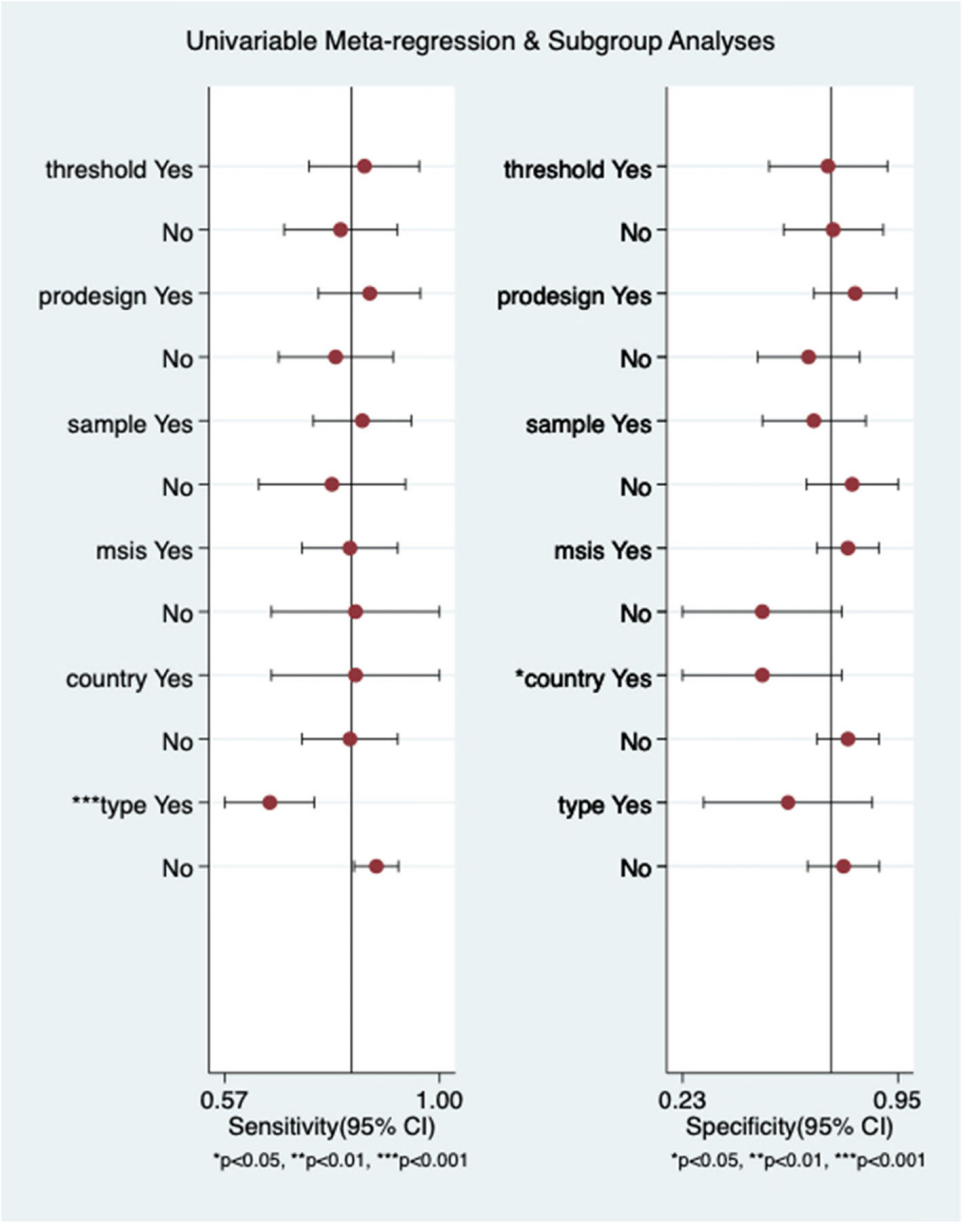
Meta-regression analysis for D-dimer with several variables

As can be seen from the forest map, there is a great heterogeneity in this study. So we conduct the following meta-regression (Fig. 8) and subgroup analysis to explore the sources of heterogeneity according to whether the threshold is the same, the study design, whether the sample size is greater than 110, the diagnostic gold standard, the country of the source of the study, and the type of sample (Table 3). Meta-regression results showed that the type of sample (serum or plasma) maybe the primary factor leading to heterogeneity of sensitivity. The country of origin of the study may be the main source of heterogeneity of specificity. The subgroup analysis results showed that the pooled sensitivity and specificity of the 4 studies with 850 ng/ml were 0.85 (95% CI, 0.69~0.93) and 0.69 (95% CI, 0.43~0.86), respectively, and the diagnostic accuracy was 0.85 (95% CI, 0.82~0.88).

**Table 3:** Subgroup analysis of D-dimer for PJI diagnosis

Subgroup analyses	No. of studies	Sensitivity (95% CI)	Specificity (95% CI)	PLN (95% CI)	NLR (95% CI)	AUC (95% CI)	Diagnostic score (95% CI)	DOR (95% CI)
Overall studies	9	0.82 (0.72–0.89)	0.73 (0.58–0.83)	2.99 (1.84–4.88)	0.25 (0.15–0.41)	0.85 (0.82–0.88)	2.50 (1.61–3.40)	12.20 (4.98–29.86)
**Study design**								
Retrospective	5	0.80 (0.63–0.90)	0.65 (0.45–0.81)	2.27 (1.35–3.84)	0.31 (0.17–0.59)	0.80 (0.76–0.83)	1.98 (0.99–2.97)	7.24 (2.68–19.53)
Prospective	4	0.86 (0.75–0.92)	0.80 (0.65–0.90)	4.34 (2.22–8.49)	0.18 (0.09–0.34)	0.90 (0.87–0.93)	3.19 (1.96–4.43)	24.39 (7.12–83.52)
**Cutoff value**								
850 ng/ml	4	0.85 (0.69–0.93)	0.69 (0.43–0.86)	2.71 (1.40–5.27)	0.22 (0.11–0.44)	0.85 (0.82–0.88)	2.50 (1.52–3.48)	12.14 (4.56–32.31)
Other	5	0.81 (0.68–0.89)	0.76 (0.60–0.87)	3.30 (1.71–6.36)	0.25 (0.13–0.51)	0.85 (0.82–0.88)	2.57 (1.25–3.88)	13.00 (3.49–48.46)
**Sample size**								
> 110	5	0.85 (0.70–0.94)	0.67 (0.44–0.84)	2.57 (1.33–4.98)	0.22 (0.09–0.52)	0.85 (0.81–0.88)	2.46 (1.10–3.81)	11.65 (3.00–45.26)
≤ 110	4	0.78 (0.66-0.86)	0.80 (0.71-0.87)	3.91 (2.48-6.17)	0.28 (0.17-0.45)	0.86 (0.83-0.89)	2.65 (1.76-3.53)	14.12 (5.82-34.29)
Serum	6	0.87 (0.78–0.92)	0.77 (0.60–0.88)	3.72 (2.09–6.62)	0.17 (0.11–0.28)	0.90 (0.87–0.92)	3.06 (2.22–3.90)	21.37 (9.21–49.63)
China	6	0.78 (0.68–0.85)	0.72 (0.62–0.81)	2.82 (1.85–4.28)	0.31 (0.19–0.49)	0.82 (0.78–0.85)	2.22 (1.35–3.08)	9.17 (3.86–21.77)
MSIS	6	0.82 (0.74–0.88)	0.78 (0.65–0.87)	3.73 (2.14–6.49)	0.23 (0.14–0.36)	0.87 (0.84–0.90)	2.78 (1.77–3.80)	16.19 (5.86–44.70)

*AUC* area under the curve of summary receiver-operating characteristic curves, *CI* confidence interval, *PLR* positive likelihood ratio, *NLR* negative likelihood ratio, *DOR* diagnostic odds ratio

## Discussion

Due to the considerable cost of treating PJI, it has caused a growing number of orthopedists to pay attention to this daunting disease [26]. As a prerequisite for determining the therapeutic regimen, an accurate diagnosis of PJI is urgent and necessary for us. As a matter of fact, it is quite arduous to accurately distinguish between aseptic loose artificial joints and PJI joints. This is because a biofilm is often formed on the surface of the prosthesis in patients with PJI, and the culture of pathogens sometimes shows negative results. Furthermore, when PJI patients present with chronic deep infection, there is no significant difference in clinical characteristics compared with aseptic loosened joints [27, 28]. It is gratifying that in recent years, an increasing number of biomarkers for the diagnosis of PJI have been found, including synovial quantitative alpha-defensin, serological white blood cell count (WBC), erythrocyte sedimentation rate (ESR) and C-reactive protein (CRP) interleukin-6 (IL-6), and procalcitonin [29]. Among them, ESR and CRP have been recommended by the American Infectious Diseases Association (IDSA) to conduct routine screening among all suspected PJI patients [30].

Recently, D-dimer has been proposed by a number of experts as a novel serum marker. D-dimer is a fibrin degradation product of disseminated intravascular coagulation and has been widely used in the diagnosis of venous thromboembolism (VTE) and infection in patients [31, 32]. According to Ribera et al., the level of synovial D-dimer is higher than normal in foal infectious joint disease [14]. The results of the study done by Bytniewski et al. show that the level of D-dimer in patients with early postoperative TJA changes faster than that of ESR and CRP, and can rise rapidly and return to normal level in a short time [33]. Subsequently, the diagnostic value of D-dimer in patients with PJI began to be valued by joint surgeons. Shahi et al. put forward the threshold of D-dimer (850 ng/ml) for the first time and considered that serum D-dimer has high sensitivity (89%) and specificity (93%). It is a valuable biomarker for the diagnosis of PJI [18]. Qin et al. used D-dimer in combination with ESR or CRP to diagnose PJI and found that combined use had higher diagnostic performance than a single test [22]. Since then, several scholars have published controversial research results.

Although a recent meta-analysis on circulating D-dimer versus fibrinogen in the diagnosis of PJI has been published, we believe that one of their included literatures is inconsistent with other subjects and should not be included in the meta-analysis. Because its research content is about D-dimer predicts persistent infection before reimplantation in two-stage exchange arthroplasty for PJI [34]. After excluding this study, they included only 5 studies that pooled the accuracy of D-dimer diagnosis. As a result, we suspect that their conclusion is unreliable. Compared with Zhang et al. [35], our meta-analysis included more studies and patients (1592 patients in 9 studies) after rigorous screening and literature quality evaluation. The overall pooled AUC of D-dimer for diagnosis PJI was 0.85 (95% CI, 0.82~0.88), which is higher than the AUC of D-dimer calculated by Zhang et al. (AUC = 0.74). As a consequence, we deem that D-dimer has a good diagnostic value in PJI, which is inconsistent with the research conclusion of Zhang et al. [35]. In addition, the pooled sensitivity and specificity were calculated to be 0.82 and 0.73 respectively. From another point of view, this is equivalent to a higher false positive rate (27%) and a lower false negative rate (18%) in D-dimer for PJI diagnosis. The LR and DOR usually indicate the effectiveness of diagnostic indicators in clinical practice [36]. A guideline defines that LR + > 2, LR − < 0.5, or DOR > 4 is considered a viable predictor, and LR + > 5, LR − < 0.2, or DOR > 10 is considered a good predictor [37]. Therefore, in terms of LR, D-dimer is a predictable index for the diagnosis of PJI, and D-dimer is a good predictive index when DOR is used as a reference parameter. Posttest probability is another parameter widely used by clinicians, including positive predictive value and negative predictive value. It reflects the probability of patients with PJI when the test results are negative or positive. The Fagan plot diagram shows that the ability of D-dimer to distinguish between PJI is general.

There is great heterogeneity in the studies of Zhang et al., but they did not provide a reasonable explanation [35]. Another advantage of this study is that we performed reasonable meta-regression and novel subgroup analysis to find the source of heterogeneity in this study. We originally considered the threshold as the main cause of heterogeneity, but the results were unexpected. In the subgroup analysis, the AUC of the same threshold group was completely consistent with the AUC of overall studies, and DOR was almost the same. We found that in the study design, the AUC and DOR of the retrospective group fluctuated downward, while the AUC and DOR of the prospective group were almost twice as high as those of all studies. Furthermore, the results of the serum group were similar with those of the prospective group. In meta-regression, the heterogeneity source of sensitivity is obviously reflected in the sample type (serum or plasma), while the specific heterogeneity source is mainly embodied in the country of study (China and USA). Therefore, we believe that the study design, sample type, and the country of study are the main factors that affect the diagnostic accuracy of D-dimer.

Admittedly, there are certain limitations in our research. First, a handful of studies have not excluded patients with inflammatory diseases, which, as mentioned above, affect D-dimer levels. Second, there is still no gold standard for the detection of PJI, and the gold standard test used in the study is only approximate. Several positive patients still miss diagnosis because the gold standard cannot detect. Third, due to the incompleteness of the original data, it is impossible to calculate the optimal cutoff value of D-dimer and conduct more detailed subgroup analysis such as the location of the joint.

## Conclusion

This study shows that D-dimer detection of PJI has a good diagnostic accuracy, but unfortunately its specificity is not high. Consequently, we believe that D-dimer is a promising biomarker for the diagnosis of PJI, which should be used in conjunction with other biomarkers or as an adjunct to other diagnostic methods to enhance diagnostic performance.

## Supplementary information

**Additional file 1.** Search Strategy.

## Data Availability

The datasets used and/or analyzed during the current study are not publicly available due to feasibility but are available from the corresponding author on reasonable request.

## Abbreviations

PJI: Periprosthetic joint infection
AAOS: The American Academy of Orthopedic Surgeon
MSIS: Musculoskeletal Infection Society
ICM: International Consensus Meeting
LR: Likelihood ratio
DOR: Diagnostic odds ratio
SROC: Summary receiver operating characteristic
AUC: Area under the curve

## References

[1] Kapadia BH (2016). Periprosthetic joint infection. Lancet.

[2] Parvizi J (2010). Periprosthetic joint infection: the economic impact of methicillin-resistant infections. J Arthroplasty.

[3] Bozic KJ (2010). The epidemiology of revision total knee arthroplasty in the United States. Clin Orthop Relat Res.

[4] Kurtz S (2007). Projections of primary and revision hip and knee arthroplasty in the United States from 2005 to 2030. J Bone Joint Surg Am.

[5] Kurtz SM (2012). Economic burden of periprosthetic joint infection in the United States. J Arthroplast.

[6] Helwig P (2014). Periprosthetic joint infection--effect on quality of life. Int Orthop.

[7] Beaule PE (2015). A protocol for a systematic review of the diagnostic accuracy of blood markers, synovial fluid, and tissue testing in periprosthetic joint infections (PJI). Syst Rev.

[8] Pozo JLD, Patel R (2009). Infection associated with prosthetic joints. N Engl J Med.

[9] Parvizi J (2011). New definition for periprosthetic joint infection: from the Workgroup of the Musculoskeletal Infection Society. Clin Orthop Relat Res.

[10] Parvizi J, Della Valle CJ (2010). AAOS Clinical Practice Guideline: diagnosis and treatment of periprosthetic joint infections of the hip and knee. J Am Acad Orthop Surg.

[11] Parvizi J, Gehrke T (2014). Definition of periprosthetic joint infection. J Arthroplasty.

[12] Hu Q, Fu Y, Tang L (2020). Serum D-dimer as a diagnostic index of PJI and retrospective analysis of etiology in patients with PJI. Clin Chim Acta.

[13] Gehrke T, Parvizi J (2014). Proceedings of the International Consensus Meeting on Periprosthetic Joint Infection. J Arthroplasty.

[14] Ribera T (2011). Synovial fluid D-Dimer concentration in foals with septic joint disease. J Vet Intern Med.

[15] Gris J (2011). Fibrin-related markers in patients with septic shock: Individual comparison of D-dimers and fibrin monomers impacts on prognosis. Thromb Haemost.

[16] Mikula T (2018). Significance of heparin-binding protein and D-dimers in the early diagnosis of spontaneous bacterial peritonitis. Mediat Inflamm.

[17] Li R (2019). Plasma fibrinogen exhibits better performance than plasma D-dimer in the diagnosis of periprosthetic joint infection: a multicenter retrospective study. J Bone Joint Surg (Am Vol).

[18] Shahi A (2017). Serum D-dimer test is promising for the diagnosis of periprosthetic joint infection and timing of reimplantation. J Bone Joint Surg (Am Vol).

[19] Fu J (2019). Synovial fluid viscosity test is promising for the diagnosis of periprosthetic joint infection. J Arthroplast.

[20] Huang J (2019). The serum level of D-Dimer is not suitable for distinguishing between prosthetic joint infection and aseptic loosening. J Orthop Surg Res.

[21] Pannu TS, et al. The utility of serum d-dimer for the diagnosis of periprosthetic joint infection in revision total hip and knee arthroplasty. J Arthroplast. 2020. 10.1016/j.arth.2020.01.034 32061477

[22] Qin L (2020). Combined measurement of d-dimer and C-reactive protein levels: highly accurate for diagnosing chronic periprosthetic joint infection. J Arthroplast.

[23] Xiong L, Li S, Dai M (2019). Comparison of D-dimer with CRP and ESR for diagnosis of periprosthetic joint infection. J Orthop Surg Res.

[24] Xu H (2019). Plasma fibrin degradation product and D-dimer are of limited value for diagnosing periprosthetic joint infection. J Arthroplast.

[25] Xie K (2017). Serum and synovial fluid interleukin-6 for the diagnosis of periprosthetic joint infection. Sci Rep.

[26] Burns A (2006). Cost effectiveness of revision total knee arthroplasty. Clin Orthop Relat Res.

[27] Parvizi J (2012). Management of periprosthetic joint infection: the current knowledge: AAOS exhibit selection. J Bone Joint Surg (Am Vol).

[28] Fitzgerald RH (1977). Deep wound sepsis following total hip arthroplasty. J Bone Joint Surg (Am Vol).

[29] Chen A, Fei J, Deirmegian C (2014). Diagnosis of periprosthetic infection: novel developments. J Knee Surg.

[30] Osmon DR, et al. Diagnosis and management of prosthetic joint infection: clinical practice guidelines by the Infectious Diseases Society of America. Clin Infect Dis. 2013;56(1). 10.1093/cid/cis803 23223583

[31] Hansrani V, Khanbhai M, Mccollum CN (2016). The diagnosis and management of early deep vein thrombosis. Adv Exp Med Biol.

[32] Chen C-J, Wang C-J, Huang C-C (2008). The value of D-dimer in the detection of early deep-vein thrombosis after total knee arthroplasty in Asian patients: a cohort study. Thromb J.

[33] Bytniewski P (2014). The dynamics of D-dimer level fluctuation in patients after the cemented and cementless total hip and total knee replacement. J Orthop Surg Res.

[34] Xu C, et al. Plasma fibrinogen may predict persistent infection before reimplantation in two-stage exchange arthroplasty for periprosthetic hip infection. J Orthop Surg Res. 2019;14. 10.1186/s13018-019-1179-9 PMC6518679 31088508

[35] Zhang Q, et al. Circulating D-dimer versus fibrinogen in the diagnosis of peri-prosthetic joint infection: a meta-analysis. Surg Infect. 2020. 10.1089/sur.2019.298 32345131

[36] Glas AS (2003). The diagnostic odds ratio: a single indicator of test performance. J Clin Epidemiol.

[37] Jaeschke R, Guyatt GH, Sackett DL (1994). Users’ guides to the medical literature: III. How to use an article about a diagnostic test: B. What are the results and will they help me in caring for my patients?. JAMA.

