# Supplementary material for: Diagnostic accuracy of D-dimer in periprosthetic joint infection: a diagnostic meta-analysis
Source: J Orthop Surg Res. 2020 Aug 17;15:334. doi: 10.1186/s13018-020-01853-w (PMC7430004; doi:10.1186/s13018-020-01853-w)
Supplement: Supplementary file 1 — Additional file 1. Search Strategy. [file 13018_2020_1853_MOESM1_ESM.docx]

Search (((Periprosthetic joint infection) OR prosthesis-related infections)) AND ((((((D-dimer) OR D-dimer fragments) OR D-dimer fibrin) OR fibrin fragment D1 dimer) OR fibrin fragment DD) OR fibrin fragment D-dimer)
